# Extracellular Vesicles as Shuttles of Tumor Biomarkers and Anti-Tumor Drugs

**DOI:** 10.3389/fonc.2014.00267

**Published:** 2014-10-08

**Authors:** Davide Zocco, Pietro Ferruzzi, Francesco Cappello, Winston Patrick Kuo, Stefano Fais

**Affiliations:** ^1^Exosomics Siena Spa, Siena, Italy; ^2^Human Anatomy Section, Department of Experimental Biomedicine and Clinical Neurosciences, Palermo and Euro-Mediterranean Institute of Science and Technology, University of Palermo, Palermo, Italy; ^3^Interferon Expression Signature Diagnostics, Cambridge, MA, USA; ^4^Anti-Tumour Drugs Section, Department of Therapeutic Research and Medicines Evaluation, National Institute of Health, Rome, Italy

**Keywords:** extracellular vesicles, exosomes, biomarkers, tumors, teranostics

## Abstract

Extracellular vesicles (EV) include vesicles released by either normal or tumor cells. EV may exceed the nanometric scale (microvesicles), or to be within the nanoscale, also called exosomes. Thus, it appears that only exosomes and larger vesicles may have the size for potential applications in nanomedicine, in either disease diagnosis or therapy. This is of particular interest for research in cancer, also because the vast majority of existing data on EV are coming from pre-clinical and clinical oncology. We know that the microenvironmental features of cancer may favor cell-to-cell paracrine communication through EV, but EV have been purified, characterized, and quantified from plasma of tumor patients as well, thus suggesting that EV may have a role in promoting and maintaining cancer dissemination and progression. These observations are prompting research efforts to evaluate the use of nanovesicles as tumor biomarkers. Moreover, EVs are emerging as natural delivery systems and in particular, exosomes may represent the ideal natural nanoshuttles for new and old anti-tumor drugs. However, much is yet to be understood about the role of EV in oncology and this article aims to discuss the future of EV in cancer on the basis of current knowledge.

## Introduction

Extracellular vesicles (EVs) are a mixed population of nanovesicles released by a variety of cells in body fluids, such as plasma, serum, or urine, of both healthy individuals and cancer patients (1, 2). EVs are classified based on their size, which ranges from 40 to 10,000 nm, biogenesis and biomarkers. Exosomes (40–100 nm) originate from the endosomal pathway and they are released by the cells following the fusion of multivesicular bodies (MVB) with the plasma membrane. Because of their origin, exosomes are enriched with scaffolding proteins (tetraspanins), proteins for endosomal trafficking (ESCRT-related proteins/Alix), and protein chaperons (heat-shock proteins). Microvesicles and apoptotic bodies (100–1000 nm) are derived by the outward budding of the plasma membrane. This mechanism enriches the membrane vesicle with phospholipids derived from the inner leaflet of the plasma membrane (e.g., phosphatidylserine). The most recently discovered EVs are large oncosomes (4000–10,000 nm), which derive from bulky protrusions of cancer cells and they are enriched with scaffolding proteins (e.g., caveolin-1) and tissue-degrading enzymes (e.g., metalloproteases). EVs have recently emerged as a promising platform for *both* diagnostic and therapeutic approaches in personalized cancer medicine. Due to their contents that include specific proteins, lipids, and nucleic acids, EVs are now considered shuttles of potential biomarkers for early detection and prognosis of either primary tumors or metastatic lesions. Additionally, EVs may carry biomarkers that are usually detected from invasive tissue biopsies, such as gene mutations for targeted cancer therapies (3). These findings suggest a new perspective for the management of cancer, utilizing EVs as a potential source of biomarkers and transitioning the field to the new concept of “liquid biopsy.” Mechanistically, EVs may transfer tumor-related molecules into non-tumoral cells to propagate the disease in both paracrine and systemic manner, or they may act as disposal systems for unwanted molecules, including anti-tumor drugs (4). Growing evidences suggest that these mechanisms may be exploited to develop new cancer vaccines and bio-inspired drug delivery systems (5, 6).

This article critically reviews recent reports on the clinical utility and current limitations of exosomes and microvesicles, generically defined as EVs, as nanoshuttles of biomarkers, anti-tumor drugs, and vaccines, opening new avenues for the clinical management of cancer.

## EVs as Shuttles of Tumor Biomarkers

### Screening and early diagnosis

Biomarkers for cancer screening and diagnosis often display low sensitivity and/or specificity, missing patients with early stage disease (false negatives) or detecting those with no disease (false positives). EVs may offer several potential benefits over current clinical biomarkers. EVs may shuttle both clinically validated biomarkers [e.g., prostate-specific antigen (PSA)] and they are a novel source of proteins and nucleic acids that could be exploited as surrogate biomarkers (7); EVs protect their cargo from the attack of nucleases and proteases, increasing biomarker half-life, and potentially facilitating sample integrity and downstream molecular analyses (8); EVs are well suited for multiplexed biomarker analyses that may increase sensitivity and/or specificity of the diagnostic assay (8, 9).

Clinical studies for EV-associated cancer biomarkers have been already described and they are summarized in Table 1. Logozzi and colleagues performed a retrospective study on EV-associated biomarkers in stage III and IV melanoma patients and they showed increased levels of caveolin-1- and CD63-positive EVs in plasma (2). EV-associated caveolin-1 displayed a sensitivity of 69% and specificity of 96.3% while levels of serum LDH were altered only in 12.5% of patients (2). Mechanistically, EVs may have a prominent role in the pathogenesis of melanoma. Melanoma cells have been shown to release exosome-associated oncoprotein MET to educate bone marrow progenitor cells and promote metastases *in vitro* and *in vivo* (10), and elevated levels of MET and phospho-MET have been detected in melanoma patients (10). Additionally, the authors showed aberrant levels of EV-associated biomarkers TYRP-2, VLA-4, HSP70, and HSP90 in the plasma of melanoma patients (10). Indeed, HSPs are emerging as another potential source of EV-based cancer biomarkers (11). HSP70 is actively secreted by different types of tumor cells through non-classical protein secretory routes, including EVs, and HSP70-positive EVs have been shown to activate macrophages (12) and natural killer cells (13–15) that act against cancer cells; while, the chaperone HSP90 has been shown to enhance cancer cell migration when is released by EV-derived cancer cells (16).

**Table 1:** **Pre-clinical and clinical studies on EV-shuttled biomarkers**.

Cancer biomarker	Disease	Indication	Biofluid	Clinical study size	Reference
PSA	Prostate cancer	Screening/early diagnosis	Urine	Controls *N* = 10; disease *N* = 24	(7)
PSA	Prostate cancer	Screening/early diagnosis	Plasma	Control *N* = 2; disease *N* = 5	(17)
EGFRvIII	Glioblastoma	Early diagnosis	Serum	disease *N* = 30	(18)
(phospho)Met	Melanoma	Early diagnosis/prognosis	Plasma	Controls *N* = 7; stage III *N* = 24; stage IV *N* = 14	(10)
Caveolin-1	Melanoma	Early diagnosis	Plasma	Controls *N* = 58; disease *N* = 90	(2)
Survivin	Prostate cancer	Early diagnosis	Plasma	HD *N* = 8; BPH *N* = 20; disease *N* = 39	(19)
CD 24	Breast cancer	Early diagnosis	Serum	HD *N* = 14, disease *N* = 18	(20)
EGRF	Lung cancer	Diagnosis/personalized medicine	Serum	HD *N* = 9; disease *N* = 9	(21)
miR-21, miR-141, miR-200a, miR-200b, miR-200c, miR-203, miR-205, miR-214	Ovarian cancer	Early diagnosis/prognosis	Serum	HD *N* = 10; stage I *N* = 10; stage II *N* = 10; stage III *N* = 20; stage IV *N* = 10	(8)
RNU6-1, miR-320, and miR-574-3p	Globlastoma	Early diagnosis	Serum	Controls *N* = 50; disease *N* = 50	(22)
TMPRSS2:ERG2 and PCA3 mRNAs	Prostate cancer	Early diagnosis	Urine	Blinded prospective study *N* = 30	(23)
let-7a, miR-1229, miR-1246, miR-150, miR-21, miR-223, and miR-23a	Colorectal cancer	Early diagnosis	Serum	Controls *N* = 22; disease *N* = 88	(23)
miR-151a-5p, miR-30a-3p, miR-200b-5p, miR-629, miR-100, and miR-154-3p	Lung cancer	Early diagnosis	Plasma	HD *N* = 10; benign disease *N* = 10; malignant disease *N* = 10	(24)
TGFB1 and MAGE3/6	Ovarian cancer	Prognosis/therapy monitoring	Plasma	HD *N* = 10; benign disease *N* = 10; malignant disease *N* = 22	(25)
TYRP2, HSP70, HSC70, VLA-4	Melanoma	Prognosis	Plasma	HD *N* = 9; stage I *N* = 2; stage III *N* = 7; stage IV *N* = 18	(10)
miR-21	Human esophageal cell carcinoma	Prognosis	Serum	HD = 41; disease *N* = 51	(26)
*KRAS*	Pancreatic cancer	Personalized medicine	Serum	HD *N* = 2; disease *N* = 2	(27)
*BRAFV600E, EGFR*	Lung cancer, melanoma	Personalized medicine	Plasma	*In vivo* model *N* = 8	(3)

EVs may be exploited as biomarker shuttles for the early diagnosis of prostate cancer (PCa). Serum PSA and prostate-specific membrane antigen (PSMA) have been found on plasma and urine-derived exosomes, though not validated in a large clinical study (7, 17). In another report, exosomal survivin was identified as promising surrogate biomarker for early diagnosis of PCa (19). Plasma levels of survivin-positive-EVs were higher in PCa patients than benign hyperplastic patients and healthy donors, potentially providing an alternative tool to reduce the number of false positives generated by the PSA test (19). Analysis of the EV cargo may allow the repositioning of a clinically validated biomarker to a new diagnostic indication. For example, the FDA has recently approved a test (commercialized by Hologic) that supports the clinical decision of repeating a biopsy in suspected PCa patients. The test is based on QRT-PCR detection of PCA3 mRNA from urine collected after digital rectal examination (DRE) and it is not recommended for the early diagnosis of PCa. Instead, a report from Dijikstra and colleagues suggested that the ratio between the levels of EV-associated PCA3 and PSA mRNAs might be useful for the early detection of PCa (9).

EVs have also been evaluated as diagnostic platforms for multiplexed approaches. Seminal work by Taylor and colleagues identified the first disease-specific miRNA signature in EVs derived from ovarian cancer patients (8). They identified a tumor-specific signature of eight miRNAs in EpCam-positive-EVs that discriminated ovarian cancer from benign ovarian disease (8). Remarkably, miRNA levels were not altered by pre-analytical variables such as collection and storage time (8). In another retrospective study, a diagnostic signature of miRNAs was found and validated in serum derived EVs from colorectal cancer patients (23). Sensitivity of the signature was higher than 90%, while serum biomarkers CEA and CA19-9 displayed sensitivities of 30.7 and 16%, respectively (23). These studies provide some level of comfort to support further research around the clinical use of EVs as biomarkers for screening and early diagnosis of cancer. However, they suffer from the lack of standardized protocols of sample collection and storage and the limited sample size (see Table 1). For the former, some general consensus was recently achieved by the International Society for Extracellular Vesicles (ISEV), though not fully implemented in clinical studies yet (28). For the latter, large regulated multi-center clinical studies are needed to validate the use of EVs for potential diagnostic applications.

### Cancer prognosis and personalized medicine

EV-based diagnostics may be a practical alternative to tumor biopsy diagnostics that are currently used for prognostic and “personalized medicine” indications. Indeed, EVs can be collected with minimally invasive procedures from a variety of body fluids; they may be more representative of the intra-tumor heterogeneity than fine needle biopsy, thus potentially revealing aggressive primary tumor features and distant metastases (25); EVs may allow the real-time monitoring of therapeutic responses and development of resistance mechanisms to targeted therapies where the mutation status is needed for patient stratification; EVs cargo may complement the use of other emerging “liquid biopsy” platforms such as circulating tumor DNA or circulating tumor cells (27). Table 1 summarizes the clinical studies on EV-based biomarker for prognosis, monitoring and personalized medicine. These studies lack the sample size and standard operating procedures required for clinical validation. However, the established presence of the disease may facilitate discovery and validation of cancer-derived biomarkers in these indications. From the regulatory perspective, we expect the initial development of EV-based laboratory developed (LDT) tests, which must meet the regulatory standard of the clinical laboratory improvement act (CLIA) and do not require to go through the FDA approval process. Though the FDA has not approved EV-based tests yet, both industrial and regulatory entities have expressed their interest in developing EV-based tests for personalized cancer medicine. There are several compelling reasons for it. EVs may represent a minimally invasive platform for the development of companion diagnostic (cDx) tests for targeted therapies. Cancer-derived EVs may be exploited for the development of blood-based cDx for cetuximab (Erbitux) since they carry both the drug target (epidermal growth factor receptor) and the mutated *KRAS* gene, which correlates with poor therapeutic responses (21, 27). Cancer-derived EVs may also shuttle genomic DNA with the mutation BRAFV600E, which may be used to develop a cDx test to identify melanoma patients eligible for the treatment with Vemurafenib (3). Moreover, personalized medicine has become one of the fastest growing segments in the molecular diagnostic market due to FDA’s recent recommendation of developing cDx tests for approval of new drugs. From the perspective of diagnostic developers, cDx tests are very attractive since they may benefit from fast-track approval and positive clinical adoption. Finally, drug developers may decide to directly reimburse the cost of cDx test to drive clinical adoption and sales of the targeted therapy, thus relieving insurances and patients from a significant economic burden.

### Technological challenges in the development of EV-based diagnostics

The field of EVs has evolved over the past 5 years especially in the technical ability to measure genomic and proteomic EV-associated biomarkers. ELISA-based technologies have been the gold standard assay for single-plexed detection of low abundant proteins (down to nanograms per milliliter levels) with high sample throughput. Conversely, multiplexed technology platforms based on mass spectrometry (MS) have been employed for proteomic analyses of cancer-derived EVs but offered rather low sample throughput and detection limits (29). Additionally, the sheer complexity of a biomolecule samples, such as blood plasma specimen further complicates any proteomic analysis. Typical blood samples can contain more than 10,000 different protein species, with concentrations varying over nine orders of magnitude. Such diversities of proteins, as well as their huge concentration ranges, present a formidable challenge for sample preparation in proteomics. Conventional protein analysis techniques, based on multidimensional separation steps and MS, fall short because of the limited separation peak capacity (up to 3000) and dynamic range of detection (~10^4^). Techniques for gene expression analysis have also their limitations. Microarrays afford high gene density and potentially high throughput but they are limited in sensitivity and dynamic range compared to RT-PCR, thus preventing discovery and analysis of many genes potentially adding power to a cancer signature.

Currently, there are four technological challenges when working with EVs: (i) the lack of standardized methods for the isolation of tumor-derived EVs. Current EV isolation protocols are largely based on non-specific physical and chemical properties, such as, size, density, and solubility as summarized in Table 2. These methods are inefficient, time consuming and costly, and produce EVs of variable in yield, purity (origin), and integrity, all features that make them poorly compatible with routine use for diagnostic purposes; (ii) the need for instrumentation capable of detecting EV biomarker panels for improved accuracy of patient diagnosis; (iii) the need for surface chemistry which enables EV biomarker detection from undiluted blood samples thereby providing maximal sensitivity; and (iv) the need for low-cost sensor platforms with high sensitivity and specificity.

**Table 2:** **Methodologies for the isolation of EVs**.

EV isolation method	Category	EV type	Reference
Ultracentrifugation	Physico-chemical	Total exosome population	(18)
Filtration (0.22 μm) and ultracentrifugation	Physico-chemical	Total exosome population	(30)
Sucrose gradient	Physico-chemical	Total exosome population	(31)
ExoQuick precipitation	Physico-chemical	Total EVs population	(32)
Immunocapture with magnetic beads with anti-EpCam antibodies	Immuno-based	EpCam-positive-EVs	(8)

## EVs as shuttles of anti-tumor drugs and cancer vaccines

Nanobiomedicine seeks to exploit the improved (and often novel) physical, chemical, and biological properties of materials at the nanometric scale with a biomimetic approach. EVs may be exploited as biomimetic nanoshuttles of drugs and cancer vaccines for several reasons (5, 33). EVs have a key role in many mechanisms promoting cancer development and progression, including cell–cell communication (34), cell migration (16), metastasis (33), angiogenesis (18), and drug resistance (5). EVs can either bind to membrane receptors or directly interact with internal compartments of the targeted cell to alter cellular behavior and their functions (35–38). Selected subpopulations of EVs or exosome-inspired biomimetic vesicles may be used to deliver anti-tumor drugs to the tumor site (4, 5). Pre-clinical and clinical studies showed that inhibition of tumor acidity induces chemosensitization in cancer patients (4, 39, 40). Since cancer exosomes act as disposal system for chemotherapic drugs, pharmacological inhibition of microenvironmental acidity may increase both exosomes targeting to the tumor site and exosomes uptake by tumor cells, by simply different electrostatic cargos (4, 39, 40). EVs may mediate gene delivery without inducing adverse immune reactions since they are amenable to autologous delivery across tissue barriers, including the blood brain barrier (41–43). Furthermore, EVs may be engineered to carry a variety of biomolecules including therapeutic RNAs (44–53), small molecules (4, 52, 53), and protein or peptide ligands (54). Finally, immune cell-derived EVs may be exploited to develop cancer vaccines. Human natural killer cell-derived exosomes have shown anti-tumor properties *in vitro* and *in vivo* (54, 55). Dendritic-derived exosomes (Dex) carry peptide-MHC complexes that can be transferred to recipient cells and express tumor-derived peptides that induce potent cytotoxic T lymphocytes (CTL)-mediated responses, leading to the regression of established tumors in mice (6, 56). Good manufacturing laboratory (GMP) procedures for exosome harvesting and purification have been set up for clinical implementation (57) and EVs have been approved for use in clinical trials with the first phase I clinical trial that started at the end of 2000, 30 months after Zitvogel’s group publication (6). In the first clinical study, patients underwent leukapheresis and exosomes were purified from monocyte derived-DC (MD-DC) in a GMP setting (58). Dex were loaded with MAGE A3 peptides, administered to 15 stage III/IV melanoma patients through four intradermal vaccinations at 1 week intervals to promote anti-tumor immunity. This study demonstrated, for the first time, the feasibility and safety of Dex-based vaccination in melanoma patients (58). In a similar clinical trial, autologous exosomes were injected weekly into 13 non-small-cell-carcinoma lung cancer (NSCLC) patients for 4 weeks but they induced weak immune responses against the tumor (59). These low immunogenic capacities have led to the development of second-generation Dex with enhanced immune-stimulatory properties (60). Viaud et al. reported the clinical grade manufacture of large-scale interferon-γ-Dex vaccines currently used in a phase II trial testing the clinical benefit of Dex as a maintenance immunotherapy in inoperable (stage IIIB to IV) NSCLC patients that responded to chemotherapy. Dex was purified from autologous maturing MD-DC loaded with MAGE-3 and NY-ESO-1, MAGE-1, MAGE-3, MART-1 restricted peptides (60). Patients received four cycles of platinum-based chemotherapy followed by a combination of a 3-week oral therapy with low dose cyclophosphamide followed by four weekly intradermal Dex injections. The study was launched in November 2009 at the Gustave Roussy and Curie Institutes and the results are not yet published (60). Another clinical study used ascites-derived exosomes (Aex) as immunomodulatory agents. In a phase I clinical trial, Aex were evaluated alone or in combination with the granulocyte-macrophage colony-stimulating factor (GM-CSF) for the immunotherapy of colorectal cancer (61). The Aex isolated by sucrose/D(2)O density gradient ultracentrifugation contained the diverse immunomodulatory markers of exosomes and tumor-associated carcinoembryonic antigen (CEA). Both therapies were safe and well tolerated with mild adverse events (grade 1–2) causally related to the use of Aex or Aex plus GM-CSF. Importantly, Aex plus GM-CSF but not Aex alone induced beneficial tumor-specific anti-tumor CTL responses (61).

Despite these promising results, the use of EVs as shuttles of anti-tumor drugs and anti-cancer vaccines is far from clinical acceptance. The lack of standardized protocols for isolation of clinical grade EVs (sub)populations and the partial understanding of the mechanisms involving EVs in cancer have so far hampered the number and the size of these clinical studies.

## Conclusion

Extracellular vesicles have the potential to revolutionize the clinical management of cancer. EVs carry a plethora of validated and surrogate biomarkers with diagnostic, prognostic, or cDx value. Among the surrogate biomarkers, molecular chaperones are likely to yield useful diagnostic and prognostic biomarkers as well as anti-cancer molecules. Despite the accumulating evidences, most of the clinical studies lack the statistical power required for biomarker validation and clinical adoption. Furthermore, the biogenesis of EVs during tumorigenesis is largely unknown, especially in the early phases of the disease. For this reason, prognosis and therapy follow up may offer a relatively easier route to biomarker validation than early diagnosis given the established presence of the disease (or absence in the case of post-surgery/treatment groups). EVs are also an intriguing source of biomarkers for personalized medicine. However, they will need to be evaluated against other “liquid biopsy platforms” such as circulating tumor DNA and circulating tumor cells, which already have established protocols for isolation from body fluids (62, 63). Detection of EV-associated biomarkers from plasma or other complex biofluids is still quite challenging since sensitive, multiplexed assays are not cost effective, robust, and high throughput enough to drive clinical adoption. Finally, several preliminary studies indicate that autologous EVs as promising delivery systems for drugs and cancer vaccines due to their ability to target the disease with only minimal side effects. More studies are warranted to test the utility of EVs as natural carriers of biomolecules for therapeutic purposes.

### 5-Year perspectives

This new area of nanobiomedicine focuses on multi-disciplinary research to build new systems for various nanobiomedical applications, ranging from the medical use of nanoplatform-based diagnostic agents, to therapeutic agents and even possible future applications of diagnosis and therapy – theranostics. One of the most important theranostic strategies is the nanoformulation of new agents based on an “all in one approach.” In this perspective, EVs can be considered potential nanocarriers of both diagnostic/prognostic biomarkers and therapeutic molecules. In the next 5 years, we expect that larger multi-center studies will be performed for the clinical validation of EV as autologous nanovectors for diagnosis, prognosis, and cancer therapy. Hopefully, these efforts will shed some light on the real value of the clinical use of EVs. We also expect EVs to have an impact in personalized cancer medicine with the introduction of cheap, multiplexed, and robust analytical assays and the growing interest of large pharmaceutical companies, regulatory agencies, and governments to reduce patient’s therapeutic burden and healthcare costs.

## Conflict of Interest Statement

The authors declare that the research was conducted in the absence of any commercial or financial relationships that could be construed as a potential conflict of interest.

## References

[1] Koga K Matsumoto K Akiyoshi T Kubo M Yamanaka N Tasaki A Purification, characterization and biological significance of tumor-derived exosomes. Anticancer Res (2005) 25(6A):3703–7 16302729

[2] Logozzi M De Milito A Lugini L Borghi M Calabrò L Spada M High levels of exosomes expressing CD63 and caveolin-1 in plasma of melanoma patients. PLoS One (2009) 4(4):e5219 10.1371/journal.pone.0005219 19381331 PMC2667632

[3] Thakur BK Zhang H Becker A Matei I Huang Y Costa-Silva B Double-stranded DNA in exosomes: a novel biomarker in cancer detection. Cell Res (2014) 24(6):766–9 10.1038/cr.2014.44 24710597 PMC4042169

[4] Federici C Petrucci F Caimi S Cesolini A Logozzi M Borghi M Exosome release and low pH belong to a framework of resistance of human melanoma cells to cisplatin. PLoS One (2014) 9(2):e88193 10.1371/journal.pone.0088193 24516610 PMC3916404

[5] Jang SC Kim OY Yoon CM Choi DS Roh TY Park J Bioinspired exosome-mimetic nanovesicles for targeted delivery of chemotherapeutics to malignant tumors. ACS Nano (2013) 7(9):7698–710 10.1021/nn402232g 24004438

[6] Zitvogel L Regnault A Lozier A Wolfers J Flament C Tenza D Eradication of established murine tumors using a novel cell-free vaccine: dendritic cell-derived exosomes. Nat Med (1998) 4(5):594–600 10.1038/nm0598-594 9585234

[7] Mitchell PJ Welton J Staffurth J Court J Mason MD Tabi Z Can urinary exosomes act as treatment response markers in prostate cancer? J Transl Med (2009) 7:4 10.1186/1479-5876-7-4 19138409 PMC2631476

[8] Taylor DD Gercel-Taylor C MicroRNA signatures of tumor-derived exosomes as diagnostic biomarkers of ovarian cancer. Gynecol Oncol (2008) 110(1):13–21 10.1016/j.ygyno.2008.04.033 18589210

[9] Dijkstra S Birker IL Smit FP Leyten GH de Reijke TM van Oort IM Prostate cancer biomarker profiles in urinary sediments and exosomes. J Urol (2014) 191(4):1132–8 10.1016/j.juro.2013.11.001 24211598

[10] Peinado H Aleckovic M Lavotshkin S Matei I Costa-Silva B Moreno-Bueno G Melanoma exosomes educate bone marrow progenitor cells toward a pro-metastatic phenotype through MET. Nat Med (2012) 18(6):883–91 10.1038/nm.2753 22635005 PMC3645291

[11] Rappa F Farina F Zummo G David S Campanella C Carini F HSP-molecular chaperones in cancer biogenesis and tumor therapy: an overview. Anticancer Res (2012) 32(12):5139–50 23225410

[12] Lancaster GI Febbraio MA Exosome-dependent trafficking of HSP70: a novel secretory pathway for cellular stress proteins. J Biol Chem (2005) 280(24):23349–55 10.1074/jbc.M502017200 15826944

[13] Vega VL Rodríguez-Silva M Frey T Gehrmann M Diaz JC Steinem C Hsp70 translocates into the plasma membrane after stress and is released into the extracellular environment in a membrane-associated form that activates macrophages. J Immunol (2008) 180(6):4299–307 10.4049/jimmunol.180.6.4299 18322243

[14] Gastpar R Gehrmann M Bausero MA Asea A Gross C Schroeder JA Heat shock protein 70 surface-positive tumor exosomes stimulate migratory and cytolytic activity of natural killer cells. Cancer Res (2005) 65(12):5238–47 10.1158/0008-5472.CAN-04-3804 15958569 PMC1785299

[15] Lv LH Wan YL Lin Y Zhang W Yang M Li GL Anticancer drugs cause release of exosomes with heat shock proteins from human hepatocellular carcinoma cells that elicit effective natural killer cell antitumor responses in vitro. J Biol Chem (2012) 287(19):15874–85 10.1074/jbc.M112.340588 22396543 PMC3346092

[16] McCready J Sims JD Chan D Jay DG Secretion of extracellular hsp90alpha via exosomes increases cancer cell motility: a role for plasminogen activation. BMC Cancer (2010) 10:294 10.1186/1471-2407-10-294 20553606 PMC3087318

[17] Mizutani K Terazawa R Kameyama K Kato T Horie K Tsuchiya T Isolation of prostate cancer-related exosomes. Anticancer Res (2014) 34(7):3419–23 24982349

[18] Skog J Würdinger T van Rijn S Meijer DH Gainche L Sena-Esteves M Glioblastoma microvesicles transport RNA and proteins that promote tumour growth and provide diagnostic biomarkers. Nat Cell Biol (2008) 10(12):1470–6 10.1038/ncb1800 19011622 PMC3423894

[19] Khan S Jutzy JM Valenzuela MM Turay D Aspe JR Ashok A Plasma-derived exosomal survivin, a plausible biomarker for early detection of prostate cancer. PLoS One (2012) 7(10):e46737 10.1371/journal.pone.0046737 23091600 PMC3473028

[20] Tanaka Y Kamohara H Kinoshita K Kurashige J Ishimoto T Iwatsuki M Clinical impact of serum exosomal microRNA-21 as a clinical biomarker in human esophageal squamous cell carcinoma. Cancer (2013) 119(6):1159–67 10.1002/cncr.27895 23224754

[21] Yamashita T Kamada H Kanasaki S Maeda Y Nagano K Abe Y Epidermal growth factor receptor localized to exosome membranes as a possible biomarker for lung cancer diagnosis. Pharmazie (2013) 68(12):969–73 24400444

[22] Manterola L Guruceaga E Gallego Perez-Larraya J A small noncoding RNA signature found in exosomes of GBM patient serum as a diagnostic tool. Neuro Oncol (2014) 16(4):520–7 10.1093/neuonc/not218 24435880 PMC3956347

[23] Ogata-Kawata H Izumiya M Kurioka D Honma Y Yamada Y Furuta K Circulating exosomal microRNAs as biomarkers of colon cancer. PLoS One (2014) 9(4):e92921 10.1371/journal.pone.0092921 24705249 PMC3976275

[24] Cazzoli R Buttitta F Di Nicola M Malatesta S Marchetti A Rom WN microRNAs derived from circulating exosomes as noninvasive biomarkers for screening and diagnosing lung cancer. J Thorac Oncol (2013) 8(9):1156–62 10.1097/JTO.0b013e318299ac32 23945385 PMC4123222

[25] Szajnik M Derbis M Lach M Patalas P Michalak M Drzewiecka H Exosomes in plasma of patients with ovarian carcinoma: potential biomarkers of tumor progression and response to therapy. Gynecol Obstet (Sunnyvale) (2013) (Suppl 4):3 24466501 10.4172/2161-0932.S4-003 PMC3899646

[26] Que R Ding G Chen J Cao L Analysis of serum exosomal microRNAs and clinicopathologic features of patients with pancreatic adenocarcinoma. World J Surg Oncol (2013) 11:219 10.1186/1477-7819-11-219 24007214 PMC3766671

[27] Kahlert C Melo SA Protopopov A Tang J Seth S Koch M Identification of double-stranded genomic DNA spanning all chromosomes with mutated KRAS and p53 DNA in the serum exosomes of patients with pancreatic cancer. J Biol Chem (2014) 289(7):3869–75 10.1074/jbc.C113.532267 24398677 PMC3924256

[28] Witwer KW Buzás EI Bemis LT Bora A Lässer C Lötvall J Standardization of sample collection, isolation and analysis methods in extracellular vesicle research. J Extracell Vesicles (2013) 2:20360 10.3402/jev.v2i0.20360 24009894 PMC3760646

[29] Duijvesz D Burnum-Johnson KE Gritsenko MA Hoogland AM Vredenbregt-van denBerg MS Willemsen R Proteomic profiling of exosomes leads to the identification of novel biomarkers for prostate cancer. PLoS One (2013) 8(12):e82589 10.1371/journal.pone.0082589 24391718 PMC3876995

[30] Gourzones C Gelin A Bombik I Klibi J Vérillaud B Guigay J Extracellular release and blood diffusion of BART viral micro-RNAs produced by EBV-infected nasopharyngeal carcinoma cells. Virol J (2010) 7:271 10.1186/1743-422X-7-271 20950422 PMC2974674

[31] Aushev VN Zborovskaya IB Laktionov KK Comparisons of microRNA patterns in plasma before and after tumor removal reveal new biomarkers of lung squamous cell carcinoma. PLoS One (2013) 8(10):e78649 10.1371/journal.pone.0078649 24130905 PMC3793941

[32] Andre F Escudier B Angevin E Tursz T Zitvogel L Exosomes for cancer immunotherapy. Ann Oncol (2004) 15(Suppl 4):iv141–4 15477298 10.1093/annonc/mdh918

[33] Hood JL San RS Wickline SA Exosomes released by melanoma cells prepare sentinel lymph nodes for tumor metastasis. Cancer Res (2011) 71(11):3792–801 10.1158/0008-5472.CAN-10-4455 21478294

[34] Iero M Valenti R Huber V Filipazzi P Parmiani G Fais S Tumour-released exosomes and their implications in cancer immunity. Cell Death Differ (2008) 15(1):80–8 10.1038/sj.cdd.4402237 17932500

[35] Andreola G Rivoltini L Castelli C Huber V Perego P Deho P Induction of lymphocyte apoptosis by tumor cell secretion of FasL-bearing microvesicles. J Exp Med (2002) 195(10):1303–16 10.1084/jem.20011624 12021310 PMC2193755

[36] Huber V Fais S Iero M Lugini L Canese P Squarcina P Human colorectal cancer cells induce T-cell death through release of proapoptotic microvesicles: role in immune escape. Gastroenterology (2005) 128(7):1796–804 10.1053/j.gastro.2005.03.045 15940614

[37] Parolini I Federici C Raggi C Lugini L Palleschi S De Milito A Microenvironmental pH is a key factor for exosome traffic in tumor cells. J Biol Chem (2009) 284(49):34211–22 10.1074/jbc.M109.041152 19801663 PMC2797191

[38] De Milito A Fais S Tumor acidity, chemoresistance and proton pump inhibitors. Future Oncol (2005) 1(6):779–86 10.2217/14796694.1.6.779 16556057

[39] Ferrari S Perut F Fagioli F Brach Del Prever A Meazza C Parafioriti A Proton pump inhibitor chemosensitization in human osteosarcoma: from the bench to the patients’ bed. J Transl Med (2013) 11:268 10.1186/1479-5876-11-268 24156349 PMC3815282

[40] Luciani F Spada M De Milito A Molinari A Rivoltini L Montinaro A Effect of proton pump inhibitor pretreatment on resistance of solid tumors to cytotoxic drugs. J Natl Cancer Inst (2004) 96(22):1702–13 10.1093/jnci/djh305 15547183

[41] Alvarez-Erviti L Seow Y Yin H Betts C Lakhal S Wood MJ Delivery of siRNA to the mouse brain by systemic injection of targeted exosomes. Nat Biotechnol (2011) 29(4):341–5 10.1038/nbt.1807 21423189

[42] Zeelenberg IS Ostrowski M Krumeich S Bobrie A Jancic C Boissonnas A Targeting tumor antigens to secreted membrane vesicles in vivo induces efficient antitumor immune responses. Cancer Res (2008) 68(4):1228–35 10.1158/0008-5472.CAN-07-3163 18281500

[43] El-Andaloussi S Lee Y Lakhal-Littleton S Li J Seow Y Gardiner C Exosome-mediated delivery of siRNA in vitro and in vivo. Nat Protoc (2012) 7(12):2112–26 10.1038/nprot.2012.131 23154783

[44] Akao Y Iio A Itoh T Noguchi S Itoh Y Ohtsuki Y Microvesicle-mediated RNA molecule delivery system using monocytes/macrophages. Mol Ther (2011) 19(2):395–9 10.1038/mt.2010.254 21102562 PMC3034851

[45] Putz U Howitt J Doan A Goh CP Low LH Silke J The tumor suppressor PTEN is exported in exosomes and has phosphatase activity in recipient cells. Sci Signal (2012) 5(243):ra70 10.1126/scisignal.2003084 23012657

[46] Hergenreider E Heydt S Tréguer K Boettger T Horrevoets AJ Zeiher AM Atheroprotective communication between endothelial cells and smooth muscle cells through miRNAs. Nat Cell Biol (2012) 14(3):249–56 10.1038/ncb2441 22327366

[47] Kosaka N Iguchi H Yoshioka Y Hagiwara K Takeshita F Ochiya T Competitive interactions of cancer cells and normal cells via secretory microRNAs. J Biol Chem (2012) 287(2):1397–405 10.1074/jbc.M111.288662 22123823 PMC3256909

[48] Mizrak A Bolukbasi MF Ozdener GB Brenner GJ Madlener S Erkan EP Genetically engineered microvesicles carrying suicide mRNA/protein inhibit schwannoma tumor growth. Mol Ther (2013) 21(1):101–8 10.1038/mt.2012.161 22910294 PMC3538300

[49] Ohno S Takanashi M Sudo K Ueda S Ishikawa A Matsuyama N Systemically injected exosomes targeted to EGFR deliver antitumor microRNA to breast cancer cells. Mol Ther (2013) 21(1):185–91 10.1038/mt.2012.180 23032975 PMC3538304

[50] Rechavi O Erlich Y Amram H Flomenblit L Karginov FV Goldstein I Cell contact-dependent acquisition of cellular and viral nonautonomously encoded small RNAs. Genes Dev (2009) 23(16):1971–9 10.1101/gad.1789609 19684116 PMC2725935

[51] Sun D Zhuang X Xiang X Liu Y Zhang S Liu C A novel nanoparticle drug delivery system: the anti-inflammatory activity of curcumin is enhanced when encapsulated in exosomes. Mol Ther (2010) 18(9):1606–14 10.1038/mt.2010.105 20571541 PMC2956928

[52] Zhuang X Xiang X Grizzle W Sun D Zhang S Axtell RC Treatment of brain inflammatory diseases by delivering exosome encapsulated anti-inflammatory drugs from the nasal region to the brain. Mol Ther (2011) 19(10):1769–79 10.1038/mt.2011.164 21915101 PMC3188748

[53] Shen B Wu N Yang JM Gould SJ Protein targeting to exosomes/microvesicles by plasma membrane anchors. J Biol Chem (2011) 286(16):14383–95 10.1074/jbc.M110.208660 21300796 PMC3077638

[54] Lugini L Cecchetti S Huber V Luciani F Macchia G Spadaro F Immune surveillance properties of human NK cell-derived exosomes. J Immunol (2012) 189(6):2833–42 10.4049/jimmunol.1101988 22904309

[55] Viaud S Terme M Flament C Taieb J André F Novault S Dendritic cell-derived exosomes promote natural killer cell activation and proliferation: a role for NKG2D ligands and IL-15Ralpha. PLoS One (2009) 4(3):e4942 10.1371/journal.pone.0004942 19319200 PMC2657211

[56] Pitt JM Charrier M Viaud S André F Besse B Chaput N Dendritic cell-derived exosomes as immunotherapies in the fight against cancer. J Immunol (2014) 193(3):1006–11 10.4049/jimmunol.1400703 25049431

[57] Lamparski HG Metha-Damani A Yao JY Patel S Hsu DH Ruegg C Production and characterization of clinical grade exosomes derived from dendritic cells. J Immunol Methods (2002) 270(2):211–26 10.1016/S0022-1759(02)00330-7 12379326

[58] Escudier B Dorval T Chaput N André F Caby MP Novault S Vaccination of metastatic melanoma patients with autologous dendritic cell (DC) derived-exosomes: results of the first phase I clinical trial. J Transl Med (2005) 3(1):10 10.1186/1479-5876-3-10 15740633 PMC554765

[59] Morse MA Garst J Osada T Khan S Hobeika A Clay TM A phase I study of dexosome immunotherapy in patients with advanced non-small cell lung cancer. J Transl Med (2005) 3(1):9 10.1186/1479-5876-3-9 15723705 PMC551593

[60] Viaud S Ploix S Lapierre V Théry C Commere PH Tramalloni D Updated technology to produce highly immunogenic dendritic cell-derived exosomes of clinical grade: a critical role of interferon-γ. J Immunother (2011) 34(1):65–75 10.1097/CJI.0b013e3181fe535b 21150714

[61] Dai S Wei D Wu Z Zhou X Wei X Huang H Phase I clinical trial of autologous ascites-derived exosomes combined with GM-CSF for colorectal cancer. Mol Ther (2008) 16(4):782–90 10.1038/mt.2008.1 18362931 PMC7106337

[62] Bettegowda C Sausen M Leary RJ Kinde I Wang Y Agrawal N Detection of circulating tumor DNA in early- and late-stage human malignancies. Sci Transl Med (2014) 6(224):224ra224 10.1126/scitranslmed.3007094 24553385 PMC4017867

[63] Yap TA Lorente D Omlin A Olmos D de Bono JS Circulating tumor cells: a multifunctional biomarker. Clin Cancer Res (2014) 20(10):2553–68 10.1158/1078-0432.CCR-13-2664 24831278

